# TFF3-dependent resistance of human colorectal adenocarcinoma cells HT-29/B6 to apoptosis is mediated by miR-491-5p regulation of lncRNA PRINS

**DOI:** 10.1038/cddiscovery.2016.106

**Published:** 2017-01-30

**Authors:** Carlos Hanisch, Jutta Sharbati, Barbara Kutz-Lohroff, Otmar Huber, Ralf Einspanier, Soroush Sharbati

**Affiliations:** 1 Department of Veterinary Medicine, Institute of Veterinary Biochemistry, Freie Universität Berlin, Berlin, Germany; 2 Lise Meitner School of Science, Berlin, Germany; 3 Institute of Biochemistry II, Jena University Hospital, Jena, Germany

## Abstract

Tumour necrosis factor-*α* (TNF-*α*) is a double-edged cytokine associated with pathogenesis of inflammatory-related cancers being also able to induce cancer cell death. In the process of tumour development or metastasis, cancer cells can become resistant to TNF-*α*. In trefoil factor 3 (TFF3) overexpressing colorectal adenocarcinoma cells (HT-29/B6), we observed enhanced resistance against TNF-*α*/interferon gamma-induced apoptosis. TFF3 is a secreted small peptide that supports intestinal tissue repair but is also involved in intestinal tumour progression and scattering. We hypothesised that TFF3 rescues intestinal epithelial cancer cells from TNF-*α*-induced apoptosis by involving regulatory RNA networks. *In silico*-based expression analysis revealed TFF3-mediated regulation of selected microRNAs as well as long non-coding RNAs (lncRNAs), whereas miR-491-5p was identified to target the lncRNA ‘psoriasis susceptibility-related RNA gene induced by stress’ (PRINS). RNA interference-based gain- and loss-of-function experiments examined miR-491-PRINS axis to exert the TFF3-mediated phenotype. Chemical inhibition of selected pathways showed that phosphatidylinositol 3-kinase/AKT accounts for TFF3-mediated downregulation of miR-491-5p and accumulation of PRINS. Moreover, we showed that PRINS colocalises with PMAIP1 (NOXA) in nuclei of HT-29/B6 possessing inhibitory effects. Immunoprecipitation experiments proved molecular interaction of PMAIP1 with PRINS. Our study provides an insight into RNA regulatory networks that determine resistance of colorectal cancer cells to apoptosis.

Impaired mucosal repair and consequently affected intestinal homeostasis seem to be the cause for a variety of intestinal diseases. Modulating effects on intestinal homeostasis and disease have been reported for human trefoil factors (TFFs). So far, three TFFs have been identified by the presence of so-called trefoil factor domains. The intestinal trefoil factor (ITF or TFF3) is known to act as a driving factor of intestinal epithelial repair.^1^ In a TNBS-induced murine colitis model, TFF3 has been shown to decrease tissue levels of tumour necrosis factor-*α* (TNF-*α*) suggesting a therapeutic potential of TFF3 in inflammatory bowel disease.^2^ Accordingly, TFF3^−/−^ mice have been shown to develop increased intestinal apoptosis. By contrast, increased expression of TFF3 has been also detected in tumours, where it appears to act as a potent mitogen and inducer of epithelial migration associated with tumour invasion, resistance to apoptosis and metastasis.^3^ Furthermore, it has been shown that TFF3 prevents TP53-dependent apoptosis of gastrointestinal cancer cell lines in a phosphatidylinositol 3-kinase (PI3K) dependent manner.^4^ Overexpression of TFF3 significantly inhibits IL-1*β* induction of TNF-*α*, this effect was reversed after pre-treatment with the PI3K/AKT inhibitor LY294002.^5^

The superfamily of TNF-proteins has pleiotropic effects.^6^ TNF-*α*-related apoptosis-inducing ligand (TRAIL) activates the extrinsic apoptosis pathway in a variety of tumour cells but not in normal cells.^7^ Interferon gamma (IFN-*γ*) has a promising role in tumour suppression by enhancing TRAIL-induced apoptosis.^8^ Moreover, a combination of IFN-*γ* and TNF-*α* has been reported to markedly sensitise metastatic colon carcinoma cells to TRAIL-induced apoptosis.^9^ Increased TRAIL receptor expression along with activation of the Wnt/*β*-catenin pathway has been associated with aggravated survival of patients with colorectal carcinoma. At the same time, increased *β*-catenin has been shown to gradually increase TRAIL receptors.^10^ Interestingly, TFF3 activates AKT by EGFR phosphorylation, which inhibits ubiquitination of *β*-catenin and consequently causes its nuclear translocation to promote proliferation of colorectal carcinoma cells.^11^ On a different note, Shiah *et al*.^12^ have shown that non-coding RNAs (ncRNAs) regulate the activator of Wnt/*β*-catenin signalling Wnt-7b. The tumour-suppressing long non-coding RNA (lncRNA) MEG3 and microRNAs (miRNAs) miR-329 and miR-410 have been reported to be downregulated in oral squamous cell carcinoma resulting in upregulation of Wnt-7b and *β*-catenin signalling.

Recent efforts in RNA biology have revealed that ncRNAs control central cellular processes such as apoptosis in gastrointestinal malignancies.^13^ Only about 10% of human RNAs are translated into functional proteins. The rest is classified into the category of ncRNAs such as the well-characterised class of miRNA or the rather heterogeneous class of lncRNAs. MiRNAs regulate gene expression via degradation of mRNAs or inhibition of translation taking part in central cellular processes.^14^ For example, direct interaction of miR-491-5p with BCL2L1 has been reported to induce apoptosis in colorectal cancer cells.^15^ Interestingly, miR-491-5p transfected hepatoma-derived Huh7 cells have been shown to suppress phosphorylation of AKT pointing to inhibition of PI3K-pathway.^16^ But also interactions between miRNAs and lncRNAs have been described, prompting the idea of regulatory networks of RNAs.^17^ Interactions between ncRNAs are suggested to provide a plethora of unprecedented regulatory mechanisms controlling development and disease. LncRNAs have been reported to be involved in several steps of cancer development as essential regulators, oncogenes or tumour suppressors.^18,19^ Along these lines, we have recently shown that the lncRNA MEG3 is a regulator of autophagy in macrophages.^20^ Another lncRNA, psoriasis susceptibility-related RNA gene induced by stress (PRINS) has been related to proliferation and differentiation states of keratinocytes. Increased expression has been described in healthy epidermis compared with psoriatic lesions.^21^ Silencing of PRINS by RNA interference (RNAi) has revealed its protective role in keratinocytes exposed to stress.^22^

TFFs are pro-invasive scatter factors interfering with PI3K/AKT, EGFR and Wnt/*β*-catenin signalling.^23^ There is increasing evidence that these pathways crosstalk in progression of cancer.^24^ In addition, several studies point out the involvement of ncRNAs in regulation of mentioned TFF-related pathways. Therefore, we speculated that TFF3 exerts its protective effects on scattering cancer cells via regulatory ncRNAs. To address this, we used TFF3-overexpressing colorectal adenocarcinoma cells (HT-29/B6/TFF3) as a model for scattering cancerous cells that are protected from IFN-*γ*/TNF-*α*-induced apoptosis. We here show that the protective phenotype is based on the interaction of miR-491-5p with the lncRNA PRINS thereby regulating the availability of the pro-apoptotic factor PMAIP1 (NOXA).

## Results

### Increased TFF3 expression mediates resistance to IFN-*γ*/TNF-*α*-induced apoptosis

**Figure 1:**
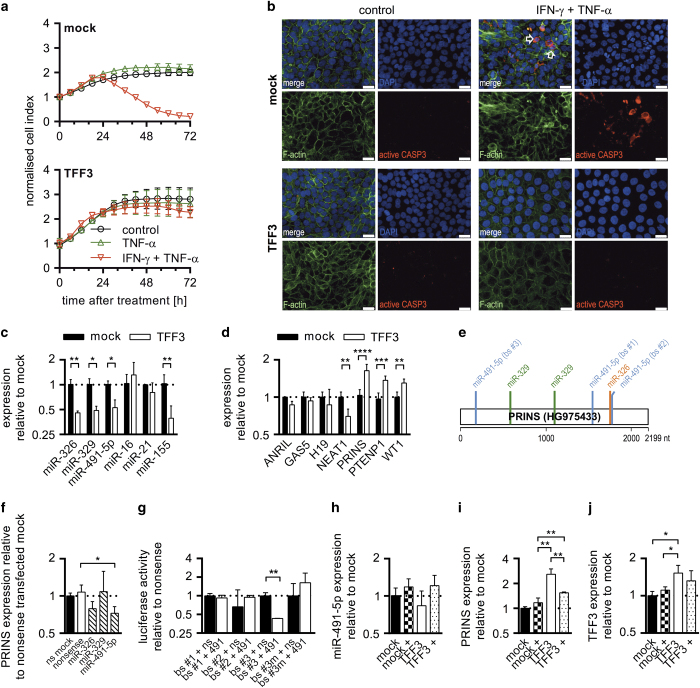
TFF3 overexpression in HT-29/B6 confers resistance to TNF-*α*/IFN-*γ*-induced apoptosis concomitant with dysregulation of apoptosis-related ncRNA. (**a**) Cell indices of HT-29/B6/TFF3 and mock-transfected colorectal adenocarcinoma cells were determined using xCELLigence analyses over 72 h. Bars show S.D. of at least four replicates. TFF3-overexpressing cells were protected from TNF-*α*/IFN-*γ*-induced apoptosis. (**b**) Apoptosis was evaluated by IF detection of active caspase-3. White arrows indicate apoptotic cells that are positive for active caspase-3 and have fragmented nuclei. Scale bars indicate 25 nm. (**c**) Expression of miRNAs was determined at day 7 after seeding. White columns show mock controls and black TFF3-overexpressing HT-29/B6 cells. Error bars indicate the S.D. Values were log2 transformed. (**d**) Expression of lncRNAs was determined at day 7 after seeding. White columns show mock controls and black TFF3-overexpressing HT-29/B6. Error bars indicate the S.D. Values were log2 transformed. (**e**) Multiple binding sites for miR-326, miR-329 and miR-491-5p were identified in the PRINS sequence (HG975433). (**f**) HT-29/B6/TFF3 were transfected with miR-326, miR-329, miR-491-5p (hatched columns) or nonsense (ns) controls (white column) and expression of PRINS was compared with ns-transfected mock (black column) 24 h post treatment. Error bars indicate the S.D. Values were log2 transformed. (**g**) Luciferase reporter gene assays identified miR-491-5p to target binding site (bs) #3. The mutated site (bs #3 m) served as a control. Samples were taken at 24 h after transfection. Black columns show ns-transfected controls. Error bars indicate the S.D. Values were log2 transformed. (**h**–**j**) Bar diagrams show the expression of miR-491-5p, PRINS and TFF3 in TNF-*α*/IFN-*γ*-treated (+) HT-29/B6/TFF3 (dotted) and mock cells (squares) compared with untreated mock controls (black) and HT-29/B6/TFF3 (white). Error bars indicate the S.D. Values were log2 transformed. Columns show means of at least three biological replicates and bars indicate S.D. *P<0.05; **P<0.01; ***P<0.001; ****P<0.0001, unpaired *t*-test.

To address our hypothesis, we applied a HT-29/B6 clone with increased TFF3 expression (HT-29/B6/TFF3, Supplementary Figure 1a) that has been shown to affect both the E-cadherin/catenin complex and tight junction proteins, as well as mock-transfected controls HT-29/B6/mock.^25,26^ Both produced highly polarised epithelial monolayers without considerable phenotypic differences (Supplementary Figure 1b). However, HT-29/B6/TFF3 exhibited slightly increased cell indices (xCELLigence) over 72 h (Figure 1a). Treatment of both clones with TNF-*α* had no obvious adverse effects on cell indices. But IFN-*γ* sensitisation followed by TNF-*α* treatment caused a marked drop of cell index at 24 h in mock-transfected controls and there was no significant change in cell index of HT-29/B6/TFF3 cells (Figure 1a). Subsequent immunofluorescence (IF) detection of active caspase-3^(ref. 27)^ and observation of fragmented and condensed nuclei proved induction of apoptosis after IFN-*γ*/TNF-*α* treatment only in HT-29/B6/mock cells (Figure 1b).

### TFF3 causes dysregulation of apoptosis-related ncRNAs

As TFF3-related pathways have been recently connected to RNA regulatory mechanisms, we hypothesised that observed TFF3-mediated resistance to apoptosis may be controlled by an interplay of miRNAs and lncRNAs. To test this, we collected a set of six miRNAs based on *in silico* analyses and literature search (Supplementary Tables 1). The collection included three well-known miRNAs (miR-16, miR-21 and miR-155) that have been commonly related to apoptotic signalling and cancer,^28–30^ as well as three underexplored miRNAs (miR-326, miR-329 and miR-491) with accumulated targets in cancer and apoptotic pathways (Supplementary Table 3). Based on our recent study,^20^ we selected lncRNAs connected to the control of apoptosis (Supplementary Table 4). Owing to the assumptions that (i) selected candidates account for the protective phenotype of TFF3-overexpressing cells and (ii) interactions between selected miRNAs and lncRNAs may exist, these two sets of ncRNAs were examined for negative correlation of expression. ncRNAs were examined in fully differentiated HT-29/B6/TFF3- and mock-transfected cells on day 7 after seeding (Figures 1c and d). Expression data during the process of differentiation on days 2 and 4 after seeding are shown in Supplementary Figures 1c–f. Downregulation of several miRNAs became apparent on day 7; miR-155, miR-326, miR-329 and miR-491 exhibited clearly (≤0.67-fold changes) and significantly decreased expression (*P*<0.05) compared with mock controls (Figure 1c and Supplementary Figures 1c and d). However, only the lncRNA PRINS showed consistent and significant upregulation over the course of differentiation (Figure 1d and Supplementary Figures 1e and f). Fully differentiated HT-29/B6/TFF3 exhibited nearly double PRINS expression levels compared with mock controls (Figure 1d, *P*<0.0001). Furthermore, PTENP1 and WT1 were significantly upregulated, whereas NEAT1 showed decreased expression (Figure 1d).

### miR-491-5p regulates cellular availability of the lncRNA PRINS

Consequently, PRINS (HG975433.1) was tested for binding sites of all four downregulated miRNAs using RNAhybrid.^31^ As shown in Figure 1e, three miR-491-5p binding sites together with two miR-329 sites and one miR-326 site were predicted (Supplementary Figure 2). To verify predictions, we transfected HT-29/B6/TFF3 with respective miRNA mimics, as well as nonsense controls. Cellular PRINS levels were significantly decreased (*P*<0.05) only after miR-491-5p transfection (Figure 1f). Interaction of miR-491-5p with predicted binding sites of PRINS was proved by means of reporter gene assays. Only co-transfection of the reporter plasmid containing binding site #3 and miR-491-5p mimic resulted in a significantly decreased luciferase activity (*P*<0.01) and a reporter plasmid including the mutated site proved the specificity of studied interaction (Figure 1g and Supplementary Figure 3).

### TFF3-dependent decrease of miR-491-5p confers PRINS-mediated resistance to apoptosis

Treatment with IFN-*γ*/TNF-*α* as described above slightly enhanced miR-491-5p expression in TFF3-overexpressing clones of HT-29/B6 cells (Figure 1h). This was accompanied with an opposite regulation of PRINS in IFN-*γ*/TNF-*α*-treated HT-29/B6/TFF3 cells compared with untreated controls, whereas PRINS levels of treated HT-29/B6/mock remained at basal levels (Figure 1i). Cytokine treatment had no effect on cellular transcript levels of TFF3 in mock and TFF3-overexpressing clones (Figure 1j).

Small interfering RNA (siRNA) against PRINS (siPRINS) was used to determine if PRINS has feedback regulatory effects on miR-491-5p. Significant knockdown of PRINS in HT-29/B6/TFF3 (*P*<0.001) did not affect cellular miR-491-5p levels (Supplementary Figures 4a and b).

**Figure 2:**
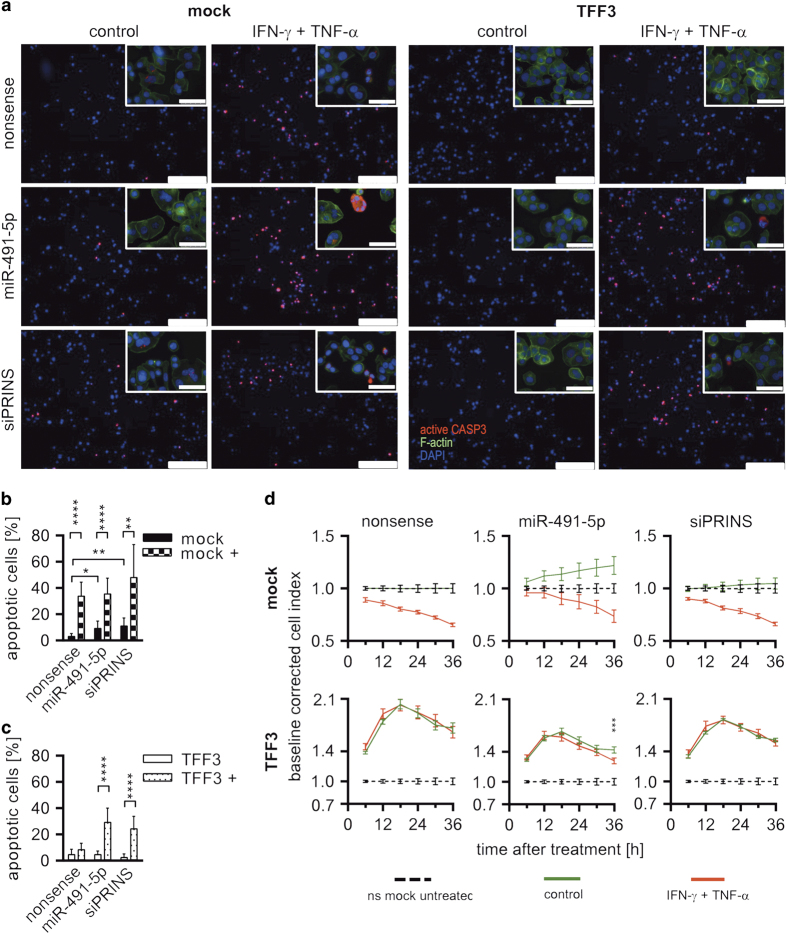
miR-491-5p-mediated PRINS regulation controls apoptosis of colorectal adenocarcinoma cells. (**a**) Apoptosis of miR-491-5p or siPRINS-transfected along with TNF-*α*/IFN-*γ* treatment was evaluated by IF detection of active caspase-3. Scale bars indicate 25 nm. Upper right section shows micrographs at higher magnification. Scale bars indicate 50 nm. (**b** and **c**) The number of apoptotic cells (positive for active caspase-3) was quantified using ImageJ considering at least four sections of three biological replicates of each treatment. Black columns show untreated mock controls and columns with squares mocks treated with TNF-*α*/IFN-*γ*. White columns show untreated HT-29/B6/TFF3 cells and dotted columns HT-29/B6/TFF3 cells treated with TNF-*α*/IFN-*γ* (+). Error bars indicate the S.D. (**d**) Cell indices of HT-29/B6/TFF3- and mock-transfected cells were determined after miR-491-5p or siPRINS transfection along with TNF-*α*/IFN-*γ* treatment by means of xCELLigence analysis over 36 h. Nonsense-transfected mock controls were selected as baseline and data were baseline corrected (cell index_sample_/cell index_mock nonsense untreated_). Bars show S.D. of at least four replicates. Values were log2 transformed. Columns show means of at least three biological replicates and bars indicate S.D. *P<0.05; **P<0.01; ***P<0.001; ****P<0.0001, unpaired *t*-test.

Next, we asked if decreased miR-491-5p and consequently increased PRINS levels are the reason for protective effects of TFF3 against IFN-*γ*/TNF-*α*-induced apoptosis in HT-29/B6. We conducted gain-of-function experiment using miR-491-5p mimics and loss-of-function experiments using siPRINS. After transfection, HT-29/B6 clones were treated with IFN-*γ*/TNF-*α* or remained untreated and apoptosis was examined by detection of active caspase-3 via IF (Figure 2a). Mocks either transfected with miR-491-5p or siPRINS exhibited a significant increase (*P*_miR-491_<0.05 and *P*_siPRINS_<0.01) of apoptotic cells (Figures 2a and b, Supplementary Figure 5). Exposure of HT-29/B6/mock to IFN-*γ*/TNF-*α* caused more than threefold, and significant (*P*<0.01) increase of apoptotic cells independent of treatment with nonsense, miR-491 or siPRINS (Figures 2a and b, Supplementary Figure 5). As observed before, nonsense-transfected HT-29/B6/TFF3 cells were protected against IFN-*γ*/TNF-*α*-induced apoptosis. But miR-491-5p transfection, as well as siPRINS along with IFN-*γ*/TNF-*α* stimulation caused a marked increase (>6-fold, *P*<0.0001) of apoptotic cells (Figures 2a and c).

Following the same treatment regime, we also examined cell indices. The xCELLigence data (Figure 2d) were baseline corrected using untreated nonsense-transfected HT-29/B6/mock (cell index_sample_/cell index_mock nonsense untreated_). Cytokine treatment caused reduction of cell index of mock cells without obvious differences among transfections. In general, cell indices of TFF3-overexpressing clones decreased after siPRINS and even more after miR-491-5p transfection compared with nonsense-transfected controls (Figure 2d). In these cells, IFN-*γ*/TNF-*α* treatment along with miR-491-5p transfection caused significantly reduced cell indices (*P*<0.001) at 36 h compared with transfected but untreated controls (Figure 2d).

### miR-491-5p-mediated regulation of PRINS depends on PI3K/AKT

**Figure 3:**
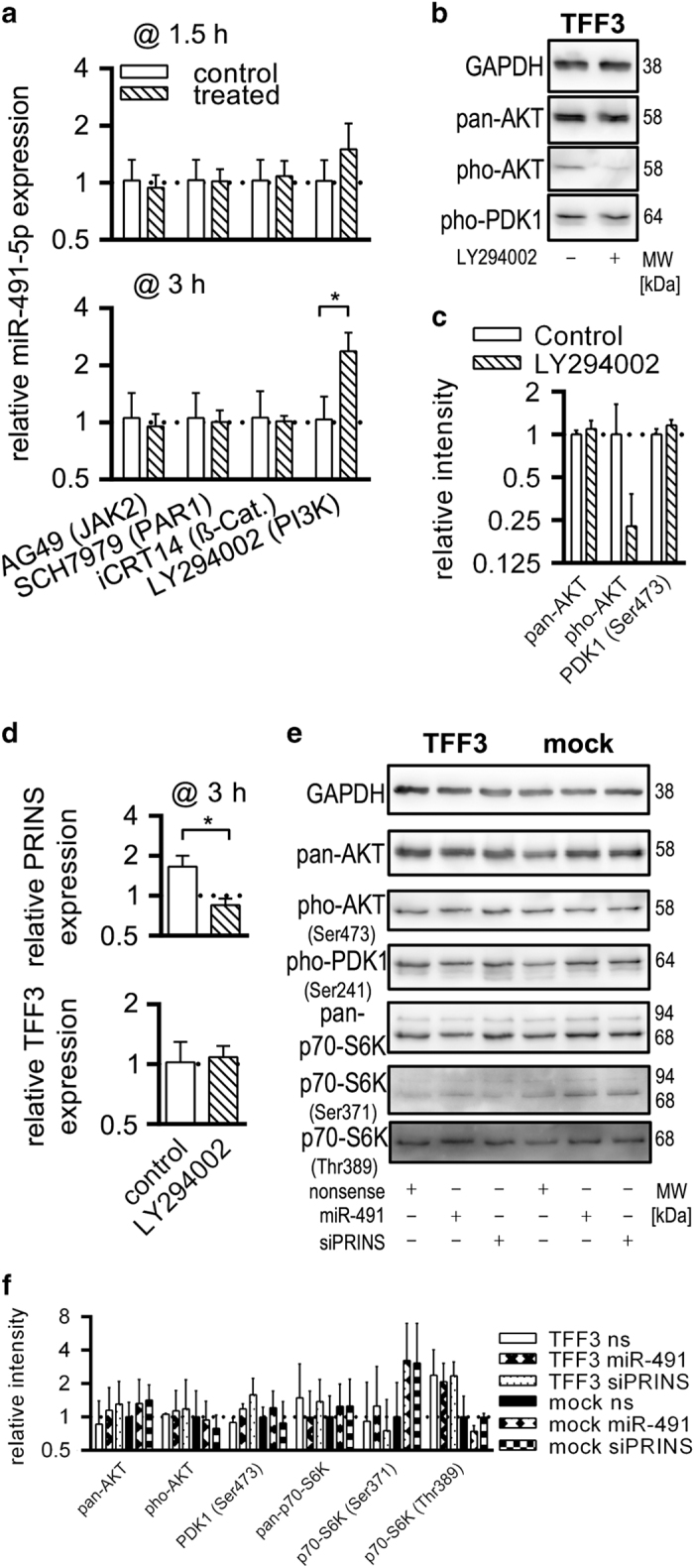
Decreased levels of miR-491-5p in TFF3-overexpressing HT-29/B6 cells depend on PI3K-AKT activation. (**a**) SMIs for relevant pathways (JAK2, PAR1, *β*-catenin and PI3K) were used to test for compensation of miR-491-5p dysregulation in HT-29/B6/TFF3 cells. White columns represent controls and hatched columns treated cells. Bars indicate the S.D. Values were log2 transformed. (**b**) Western blotting proved the functionality of the PI3K inhibitor in HT-29/B6/TFF3 cells. Black borders indicate cropped area of the western blot. Molecular weights (MWs) were determined with the Fusion Capt Advance Software (Vilber Lourmat) according to the MW marker Page Ruler Plus Prestained Protein Ladder (Thermo Fisher Scientific GmbH). (**c**) Densitometric analysis of **b** signals was performed using the Fusion Capt Advance Software (Vilber Lourmat, Eberhardzell, Germany). Columns show normalised means (protein of interest/GAPDH) of three independent replicates and bars represent the S.D. Values were log2 transformed. (**d**) PI3K inhibition decreased cellular levels of PRINS and had no effect on TFF3 levels. Values were log2 transformed. (**e**) Western blot detection of phosphorylation status of AKT, PDK1 and p70-S6K upon miR-491-5p or siPRINS transfection were determined. Black borders indicate cropped area of the western blot. MWs were determined with the Fusion Capt Advance Software (Vilber Lourmat) according to the MW marker Page Ruler Plus Prestained Protein Ladder (Thermo Fisher Scientific GmbH). (**f**) Densitometric analysis of section** e** was performed using the Fusion Capt Advance Software (Vilber Lourmat). Columns show normalised means (protein of interest/GAPDH) of three independent replicates and bars represent the S.D. Values were log2 transformed.

We used small molecule inhibitors (SMI) to examine signalling pathways that exert miR-491-5p inhibitory effect of TFF3. Applied SMIs are known to inhibit TFF3-relevant pathways (JAK2, PAR1, *β*-catenin and PI3K). As shown in Figure 3a, inhibition of PI3K/AKT by LY294002 resulted in increased miR-491-5p expression as early as 1.5 h after treatment. At 3 h, miR-491-5p level was more than twofold and significantly increased (*P*<0.05) compared with control-treated HT-29/B6/TFF3. Western blotting revealed efficient inhibition of AKT after LY294002 treatment of HT-29/B6/TFF3 without effects on PDK1 (Ser241) phosphorylation (Figures 3b and c). Chemical PI3K/AKT inhibition and consequently increased miR-491-5p efficiently reduced cellular PRINS levels compared with untreated HT-29/B6/TFF3 cells without effects on TFF3 expression (Figure 3d). To determine whether miR-491-5p regulation of PRINS had feedback effects on PI3K/AKT signalling, we examined activation states of downstream kinases after RNAi (siPRINS and miR-491 mimic). RNAi had no obvious effect on phosphorylation status of AKT and PDK1. p70-S6K Thr389 phosphorylation was diminished in HT-29/B6/mock compared with TFF3-overexpressing cells (Figures 3e and f). SiPRINS as well as miR-491-5p enhanced phosphorylation of p70-S6K Ser371 in mock cells compared with nonsense-transfected controls (Figures 3e and f).

### PRINS interacts with PMAIP1 (NOXA) in nuclei of HT-29/B6/TFF3 cells and determines its availability

**Figure 4:**
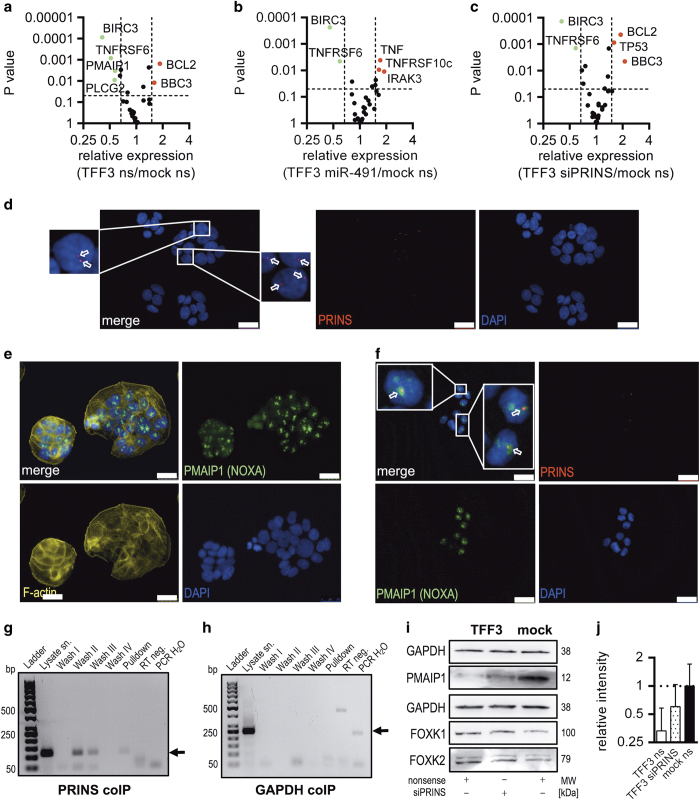
PRINS interacts with PMAIP1 and determines its availability. (**a**–**c**) Volcano plots show significantly dysregulated apoptosis-relevant genes in nonsense, miR-491-5p or siPRINS-transfected HT-29/B6/TFF3 cells compared with nonsense-transfected mock controls (green: significantly downregulated; red: significantly upregulated). (**d**) PRINS localisation was determined by Stellaris FISH, arrows show focal PRINS signals (red: PRINS, blue: DAPI, scale bars indicate 25 nm). (**e**) PMAIP1 localisation was determined by IF (green: PMAIP1, yellow: f-actin, blue: DAPI, scale bars indicate 25 nm). (**f**) Colocalisation of PRINS with PMAIP1 was determined by combination of FISH and IF (red: PRINS, green: PMAIP1, blue: DAPI, scale bars indicate 25 nm). (**g** and **h**) Co-IP using the PMAIP1-specific antibody (CST, #D8L7U), experiments show specific interaction of PRINS with PMAIP1 proved by PCR (pulldown sample). Lysate supernatant (sn) and diverse washes were taken as controls. GAPDH serves as control for assay specificity. (**i**) PRINS knockdown in HT-29/B6/TFF3 cells causes accumulation of PMAIP1 with no differences in FOXK1 and 2. Black borders indicate cropped area of the western blot. MWs were determined with the Fusion Capt Advance Software (Vilber Lourmat) according to the MW marker Page Ruler Plus Prestained Protein Ladder (Thermo Fisher Scientific GmbH). (**j**) Densitometric analysis of section i was performed using the Fusion Capt Advance Software (Vilber Lourmat). Columns show normalised means (protein of interest/GAPDH) of three independent replicates and bars represent the S.D. Values were log2 transformed.

It has been recently shown that FOXK2-dependent induction of apoptosis results in unexpectedly increased phosphorylation of p70-S6K, as well as upregulation of pro-apoptotic genes such as PMAIP1 (NOXA).^32^ We used an RT-qPCR array comprising genes relevant to apoptosis^33^ and evaluated the effect of miR-491-5p mimics, as well as siPRINS. TFF3 overexpression caused increased cellular BCL2 and BBC3 levels. The pro-apoptotic factor PMAIP1 (NOXA), death receptor TNFRSF6 (FAS), growth factor-related phospholipase PLCG2, as well as BIRC3 were significantly downregulated in nonsense-transfected HT-29/B6/TFF3 cells (Figure 4a). Transfection of HT-29/B6/TFF3 cells with miR-491-5p mimic compensated for BCL2, BBC3, PLCG2 and PMAIP1 dysregulation (Figure 4b). However, treatment of HT-29/B6/TFF3 cells with siPRINS shifted only PLCG2 and PMAIP1 expression to mock levels suggesting functional coherence to PRINS (Figure 4c).

Consequently, we focused on PMAIP1 as a prominent pro-apoptotic factor. We first determined cellular localisation of PRINS and PMAIP1 in HT-29/B6/TFF3 cells. Using Stellaris RNA fluorescence *in situ* hybridisation (FISH) probes, PRINS was exclusively detected in nuclei (Figure 4d). In most cells, only few foci per nucleus were detected. Negative controls proved the specificity of signals (Supplementary Figures 6a and b). Cellular distribution of PMAIP1 in TFF3-overexpressing cells was investigated by means of IF. Similar to PRINS, a small number of accumulated signals per nucleus were detected (Figure 4e). Colocalisation studies revealed perfectly matching focal fluorescence signals of PRINS and PMAIP1 in nuclei (Figure 4f). As FOXK2 has been recently related to PMAIP1, we also examined colocalisation of PRINS with FOXK1 and FOXK2. However, no spatial coherence between PRINS and FOXK proteins was observed (Supplementary Figures 6c–f). Colocalisation of PRINS and PMAIP1 prompted their molecular interaction, which was addressed by co-immunoprecipitation (co-IP) of PRINS after pulldown of PMAIP1. As shown in Figure 4g, PRINS was detected in lysates used for co-IP. Wash steps with increased stringency resulted in successively decreased detection of PRINS. In RNA samples isolated from supernatants of the final wash PRINS was no longer detectable (Figure 4g, wash IV). However, in RNA samples extracted after release of PMAIP1 from beads, PRINS was still detectable, pointing to protection from RNase digestion by specific binding either directly to PMAIP1 or to ribnucleoprotein complexes including PMAIP1 (Figure 4g, pulldown). GAPDH served as a control for co-IP and disappeared fully after RNase digestion (Figure 4h). Another control for specificity of co-IP was considered by replacing the PMAIP1-specific antibody with normal rabbit IgG. PRINS detection was decreased with enhanced washing and no PRINS was detected in pulldown samples (Supplementary Figure 6g). Furthermore, we addressed regulatory effects of PRINS on PMAIP1. For this purpose, we transfected HT-29/B6/TFF3 cells with siPRINS and studied PMAIP1 (NOXA) levels by western blotting. As shown in Figures 4i and j, PRINS knockdown clearly enhanced cellular PMAIP1 levels in TFF3-overexpressing cells compared with nonsense-transfected controls. PRINS knockdown had no obvious effects on FOXK1 and 2 expression levels (Figure 4i).

## Discussion

Restitution of intestinal tissue and maintenance of homeostasis requires migration of intact cells at the site of injury. TFF3 seems to take a key role in this process not only as a potent mitogen but also by inhibiting apoptosis when epithelial cells lose their integrity to migrate to the site of injury. However, pathologic TFF3 expression exerts similar effects in different cancers and promotes metastatic seeding.^25,34^ Human intestinal adenocarcinoma cells HT-29/B6 used in this study grow as polarised monolayers and represent a well-established *in vitro* model for intestinal cell biology.^35^ Several studies suggested a close connection of TFF3 to PI3K/AKT and Wnt pathways, both commonly reported to be dysregulated in cancer. But so far little is known about the molecular mode of TFF3 action. Although the role of PI3K/AKT in metastatic colon cancer has been recognised long ago, it became recently apparent that ncRNAs are potent regulators in this context.^36^ The importance of ncRNAs is demonstrated by the fact that a substantial part of genome is devoted to their transcription. In this context, RNA–RNA interactions have been shown to regulate wide-ranging cellular processes. As the power of miRNA in regulation of mRNA has been explored elaborately, we are beginning to recognise the complex interactions of miRNAs and lncRNAs and ensuing RNA regulatory networks. Interaction of miRNAs with lncRNAs is an emerging scientific field helping to disclose missing links in basal cellular regulation helping to provide novel therapeutic strategies in diverse diseases. We considered TFF3-mediated resistance to apoptosis in colorectal adenocarcinoma cells as a basis for exploring RNA regulatory networks that confer the observed apoptosis-resistant phenotype.

Consequently, we considered ncRNAs based on our earlier studies, literature searches and *in silico* analyses. To test for interactions between miRNAs and lncRNAs, contrary expression was examined. Expression analyses were performed along the course of differentiation on days 2, 4 and 7 after seeding. As TFF3 is constitutively overexpressed in HT-29/B6 cells, we hypothesised that potential RNA regulatory networks that exert TFF3 effects exist regardless of the differentiation state. Therefore, we focused primarily on candidates that showed opposing expression throughout differentiation period. We concentrated on miR-155, miR-326, miR-329 and miR-491-5p showing stable downregulation after prolonged incubation. PRINS was the only lncRNA fulfilling the criterion of contrary expression with respect to dysregulated miRNAs and the criterion of possessing a stable dysregulation. However, the expression kinetics indicated that regulation of PRINS cannot solely be attributed to TFF3. This suggests the assumption that differentiation state of cells may have an influence on the expression of relevant genes and justifies the 4-day pre-incubation of HT-29/B6 subclones in experiments. Our results show that only miR-491-5p significantly regulates PRINS. After transfection with miR-326 mimic, we recorded a trend in PRINS downregulation. Nevertheless, regulation of PRINS by miR-326 may be anticipated. Splicing variants and alternative tertiary structures of PRINS may appear in a differentiation-dependent manner, which could be under the control of miRNAs other than miR-491-5p. Future studies are needed to disclose alternative PRINS architecture.

Our results show that TFF3 inhibits miR-491-5p expression in HT-29/B6 cells thus reducing cellular levels of PRINS. As miR-491-5p has been reported to be a pro-apoptotic molecule,^37,38^ we assumed that protective effects of TFF3 rely on miR-491-5p-dependent accumulation of PRINS. Interestingly, 3 h after cytokine stimulation miR-491-5p tended to be increased in HT-29/B6/TFF3 cells. On the contrary, PRINS expression significantly decreased in HT-29/B6/TFF3 cells, whereas expression levels remained unchanged in HT-29/B6/mock-transfected cells. PRINS expression in HT-29/B6/mock cells is presumably at a consistently low basal level, so that a further decrease is not anticipated. Cytokine stimulation influenced the observed miR-491-5p-PRINS axis. Moreover, gain-of-function as well as loss-of-function experiments pushed HT-29/B6/TFF3 phenotype toward mock controls proving that the observed protective effects of TFF3 rely on the mentioned axis. MiR-491-5p has been reported to efficiently induce apoptosis in ovarian carcinoma by targeting BCL2L1 and accumulating BCL2L11 in its dephosphorylated form.^37^ In pancreatic cancer, miR-491-5p expression was decreased compared with normal tissue. It has been shown to target BCL2L1 and TP53, in consequence cancer cell proliferation was decreased with increased doses of miR-491-5p.^38^ As mentioned above, disease-related regulation of apoptotic protein-coding transcripts via miRNAs is a well-recognised fact. Here, we show that miRNA-mediated regulation of lncRNAs such as PRINS constitutes a further level of regulation of apoptosis in colorectal cancer cells. A class of lncRNAs has been defined as competing endogenous RNAs that vie for miRNA-binding sites with a protein-coding target thus functioning as a miRNA sponge.^39^ In our study, TFF3-dependent downregulation of miR-491-5p caused accumulation of PRINS. However, specific knockdown of PRINS had no effects on miR-491-5p levels. Therefore, it is unlikely that PRINS functions as a miRNA sponge.

TFF3-deficient mice have been reported to exhibit increased colonocyte apoptosis and exogenous TFF3 has been shown to protect from apoptosis. The anti-apoptotic effects of TFF3 have been related to PI3K signalling and TP53 inhibition.^4^ In miR-491-5p mimics- as well as siPRINS-experiments, we showed that HT-29/B6/TFF3 cells became more susceptible to apoptosis. Unexpectedly, both treatments caused increased phosphorylation of p70-S6K in HT-29/B6/mock cells. In a recent study, it has been shown that knockdown of FOXK2 impaired proliferation and induced apoptosis. In accordance with our observations, induction of apoptosis was accompanied by unexpectedly increased phosphorylation of p70-S6K. The authors have speculated that enhanced p70-S6K phosphorylation in apoptotic cells may represent a compensatory mechanism. At the same time, they reported that absence of FOXK2 leads to upregulation of pro-apoptotic PMAIP1.^32^ Consequently, we screened for protein-coding transcripts that are relevant to apoptotic signalling^33^ and are compensated upon restoring miR-491-5p deficiency, as well as PRINS excess. Thereby identified PLCG2 and PMAIP1 are apparently controlled by the examined miR-491-5p-PRINS axis. PLCG2 takes part in protein kinase C (PKC) signalling and has been shown to cause nuclear translocation of PKC*α* during the induction of apoptosis in gastric cancer cells.^40^ Our localisation studies showed that PRINS and PMAIP1 (NOXA) exhibit focal and overlapped fluorescence signals in the nucleus prompting their functional interaction, which we proved by co-IP experiments. Among others, lncRNAs have been described to function as scaffolds that bring together different proteins to build ribonucleoprotein complexes.^41^ Our data point out that PRINS interacts either directly or presumably via involved ribonucleoprotein complexes with PMAIP1 in the nucleus. We also showed that PRINS knockdown resulted in PMAIP1 accumulation. PMAIP1 is known to recruit MCL1 from the cytosol to the mitochondria causing its degradation.^42^ Colorectal tumours have been described to show both nuclear and cytoplasmic expression of PMAIP1.^43^ However, its function in the nucleus is not yet known. We assume that PRINS functions as a scaffold that ascertains nuclear localisation of PMAIP1 retaining its apoptotic action in the cytoplasm/mitochondria.

In conclusion, TFF3 exerts its anti-apoptotic effects by means of AKT/PI3K-dependent miR-491-5p downregulation and increased levels of PRINS in HT-29/B6 cells. PRINS interacts with PMAIP1 in the nucleus and presumably prevents its pro-apoptotic action in the cytoplasm. Our findings will help to gain knowledge about the molecular mechanisms behind resistance of cancer cells to apoptosis and show that RNAi-based approaches may contribute to the development of more effective TRAIL-based cancer therapies as explored in colorectal adenocarcinoma cells.

## Materials and Methods

### Cell culture, xCELLigence, chemical treatment and nucleofection

The subclone HT-29/B6 ^(ref. 44)^ of the human colorectal adenocarcinoma cell line HT-29 (ACC299), and its stably transfected clones HT-29/B6/TFF3 and HT-29/B6/mock^25^ were maintained in RPMI supplemented with 10% (v/v) FCS superior (Biochrom, Berlin, Germany) and 400 *μ*g/ml G418 (Carl Roth, Karlsruhe, Germany)—for stably transfected clones—at 37 °C and 5% CO_2_ in 75 cm^2^ flasks (Sarstedt, Nümbrecht, Germany). HeLa cells (ATCC, Wesel, Germany, CCL-2) were cultured in RPMI supplemented with 10% (v/v) FCS superior, gentamicin (Biochrom) and l-glutamine (Biochrom). Cells were passaged when reached 70–90% confluence.

The xCELLigence (ACEA Biosciences, San Diego, CA, USA) system was used according to the instructions of the supplier. Cells were seeded at a density of 1.5×10^4^ per well in a gold cell sensor incorporated E-plate. Physiological changes of cells were analysed by the electronic impedance, which is represented by the unit-less cell index. Cell index was monitored every 15 min for a period of up to 7 days after treatment. Four days after incubation, cells were sensitised using 40 ng/ml IFN-*γ* (CST, Frankfurt, Germany) for 15 min. After removing the media, cells were treated with 100 ng/ml TNF-*α* (Sigma Aldrich, Taufkirchen, Germany). Untreated controls were considered. Cell indexes were normalised immediately after treatment.

For SMI experiments, cells were treated 4 days after seeding with 50 *μ*M AG490, 1 *μ*M SCH7979, 50 *μ*M iCRT14, 20 *μ*M LY294002 (all provided by Tocris Bioscience, Wiesbaden, Germany), respectively. Samples were taken at 1.5 and 3 h after treatment.

HT-29/B6 derivatives were transfected using the Nucleofector Technology (Lonza AG, Köln, Germany). For nucelofection, 1×10^6^ cells were used together with 50 pmol of miRVana miRNA mimics (Life Technologies, Darmstadt, Germany), 100 pmol siRNA *versus* PRINS (5′- UUUCUGGAAUGAUGUCCAA-3′, Eurofins Genomics, Ebersberg, Germany) or 50–100 pmol nonsense control RNA (Thermo Fisher Scientific GmbH, Darmstadt, Germany), respectively. Twenty-four hours after transfection, cells were washed with PBS and lysed for RNA or protein isolation.

### RNA isolation and RT-qPCR

For expression analysis, 5×10^5^ cells per well were plated and grown on six-well plates (Sarstedt) for 4 days or taken after 24 h after nucleofection. Total RNA was isolated with miRVana Isolation Kit (Life Technologies) and quality controlled as described earlier.^45^ MiRNA as well as lncRNA-specific RT-qPCR was performed as described previously^20,45,46^ considering SNORD44 and 47 as references for miRNA and GAPDH, SDHA and SNORD47 for lncRNA, respectively.

### Dual luciferase reporter assays

To analyse the interaction between miR-491-5p and predicted binding sites in PRINS (HG975433), dual luciferase reporter assays (Gaussia/Cypridina NEB, Frankfurt, Germany) were performed using the vectors pTKGluc and pTKCluc (NEB) together with synthetic binding sites (Supplementary Figure 2) as described previously.^47^ For transfection, 1 *μ*g of pTKGluc derivatives, 100 ng pTKCluc (for normalisation) and 100 pmol miRNA mimic were used.

### IF detection and western blot

Cells were seeded (5×104 cells per well) on eight-well tissue culture chamber slides (Sarstedt). After attachment, monolayers were treated with 40 ng/ml IFN-*γ* for 15 min, washed with PBS and exposed to 100 ng/ml TNF-*α* or remained untreated as controls. Twenty-four hours after treatment, cells were washed with PBS and IF of active caspase-3 was performed as described earlier^27^ and signals were detected using the microscope DMI6000 B (Leica, Wetzlar, Germany). At least four randomly selected sections were taken for semi-automatic cell counting with ImageJ.^48^ Numbers of DAPI-stained nuclei and active caspase-3-positive cells were used to calculate the percentage of apoptotic cells. At least three biological replicates were considered. FOXK1, 2 or PMAIP1 (NOXA) were detected by means of IF using anti-FOXK1 (1 : 400, CST #12025), anti-FOXK2 (1 : 400, CST #12008) or anti-NOXA (1 : 400, CST, #D8L7U) diluted in 1% BSA in PBS.

Western blotting was performed as described earlier^20,49^ using following antibodies and dilutions: GAPDH, 1 : 2000 (CST # D16H11); pho-AKT, 1 : 2000 (Ser473, CST #D9E); pan-AKT, 1 : 1000 (CST #C67E7); pho-PDK1, 1 : 1000 (Ser241, CST #C49H2); pan-P70-S6K, 1 : 1000 (CST #49D7), pho-P70-S6K, 1 : 1000 (Ser371, CST #9208); pho-P70-S6K, 1 : 2000 (Thr389, CST #108D2); NOXA, 1: 750 (CST, #D8L7U), FOXK1, 1 : 1000 (CST #12025); FOXK2, 1 : 1000 (CST #12008). HRP-labelled anti-rabbit IgG (CST #7074P2) was used at a concentration of 1 : 2000.

### Fluorescence *in situ* hybridisation

In all, 5×10^4^ cells per well of an eight-well chamber slide (Sarstedt) were seeded incubated for 4 days. PRINS localisation in HT-29/B6 and its subclones were performed by means of custom Stellaris RNA FISH System (Biosearch Technologies, Petaluma, CA, USA) according to the manufacturers protocol. For this purpose, 35 PRINS-specific probes were used which were tagged with CAL Fluor Red 610 (Supplementary Table 5).

### Combined FISH and IF for RNA-protein colocalisation studies

Cells were seeded as described above. After removing media, cells were washed with PBS and fixed with 3.7% formaldehyde at room temperature for 10 min. Monolayers were washed twice with PBS for 5 min and permeabilised using 0.1% (v/v) Tween 100 and 1% (w/v) BSA in PBS for 30 min. Cells were incubated with the primary antibody (diluted in PBS with 1% (w/v) BSA) either for 1 h at room temperature or overnight at 4 °C. After washing twice with PBS for 5 min, the secondary antibody anti-rabbit 488 (Thermo Fisher Scientific GmbH (#35552) goat anti-rabbit IgG (H+L) Dylight 488 conjugated) was applied using a dilution of 1 : 200 in in PBS with 1% (w/v) BSA. Cells were washed twice with wash buffer from Stellaris RNA FISH System (Biosearch Technologies). Hybridisation and detection of PRINS was performed as described above. Nuclei were detected using 0.5 *μ*g/ml DAPI for 4 min and F-actin was detected using 2.5 U/ml phalloidin (Atto 647N #65906, Sigma Aldrich) for 20 min.

### Immunoprecipitation and RNA pulldown

Immunoprecipitation was performed as described earlier^47^ with following modifications. To address PRINS interaction with PMAIP1, 8×10^6^ HT-29/B6/TFF3 cells were seeded in 75 cm^2^ flasks and incubated for 4 days. After washing three times with PBS, monolayers were lysed using 750 *μ*l of cell lysis buffer (CST) supplemented with 1 mM PMSF (Sigma Aldrich). After 5-min incubation on ice, cells were scraped off and lysates were transferred into a tube for three rounds of sonication using a Branson Sonifier 250 (5 s, constant duty cycle, output control 1). The lysate was centrifuged for 10 min at 14 000×*g* and the supernatant was precleared using 40 *μ*l Protein G Magnetic Beads (NEB). For this purpose, 200 *μ*l of lysate was incubated with prewashed (Cell Lysis Buffer, CST) magnetic beads for 2 h at 4 °C and gentle rotation. The precleared lysate was incubated overnight at 4 °C with anti-PMAIP1 antibody (CST, #D8L7U) using a 1 : 100 dilution. PMAIP1 was immunoprecipitated for 4 h at 4 °C using 40 *μ*l of prewashed magnetic beads. The supernatant was taken as a control sample (Figure 4i, lysate sn.). To remove unspecifically binding RNA, bead-bound ribonucleoprotein complexes were prone to RNase A digestion for 1 h at 37 °C using 20 ng/ml RNase A and RNase-buffer (50 mM Tris-HCl, 10 mM EDTA, pH 8). Digested RNAs that were not bound to protein were removed by means of four successive washes of beads with increasing stringency at room temperature and with gentle rotation. Wash I: 5 min using cell lysis buffer (CST). Wash II and III: 5 min using Stellaris Wash Buffer A. Wash IV: 5 min using Stellaris Wash Buffer B. The supernatants were kept as controls (Figure 4i, Washes I–IV). After washing steps, complexes bound to the beads were resuspended in cell lysis buffer (CST) and treated with 200 *μ*g/ml proteinase K for 30 min at 37 °C to dissociate specifically protein-bound RNAs that were protected from RNase A digestion. Released nucleic acids were precipitated by ethanol and the pellet was resolved in 300 *μ*l miRVana lysis buffer to extract the RNA with the miRVana Isolation kit (Life Technologies). Isolated RNA was reverse transcribed and PMAIP1-bound PRINS was detected as described above. GAPDH mRNA was considered as a soluble and not PMAIP1 associated control. For proving the specificity of the assay, the PMAIP1-specific antibody was replaced with same amounts (1.1 *μ*g) of normal rabbit IgG (Merck Chemicals GmbH, Darmstadt, Germany).

### Statistical analysis

All experiments were performed considering at least three biological replicates. Unpaired *t*-tests were performed using GraphPad Prism Version 4.03 for Windows (GraphPad Software, La Jolla, CA, USA; www.graphpad.com). Asterisks in figures summarise *P*-values (*P<0.05; **P<0.01; ***P<0.001; ****P<0.0001).
