# Supplementary material for: TFF3-dependent resistance of human colorectal adenocarcinoma cells HT-29/B6 to apoptosis is mediated by miR-491-5p regulation of lncRNA PRINS
Source: Cell Death Discov. 2017 Jan 30;3:16106–. doi: 10.1038/cddiscovery.2016.106 (PMC5279457; doi:10.1038/cddiscovery.2016.106)
Supplement: Supplementary Information [file cddiscovery2016106-s1.pdf]

TFF3-dependent resistance of human colorectal adenocarcinoma cells HT-29/B6 to apoptosis is mediated by miR-491-5p regulation of lncRNA PRINS

Carlos Hanisch[1], Jutta Sharbati[1], Barbara Kutz-Lohroff[1], Otmar Huber[2], Ralf Einspanier[1], Soroush Sharbati[1*]

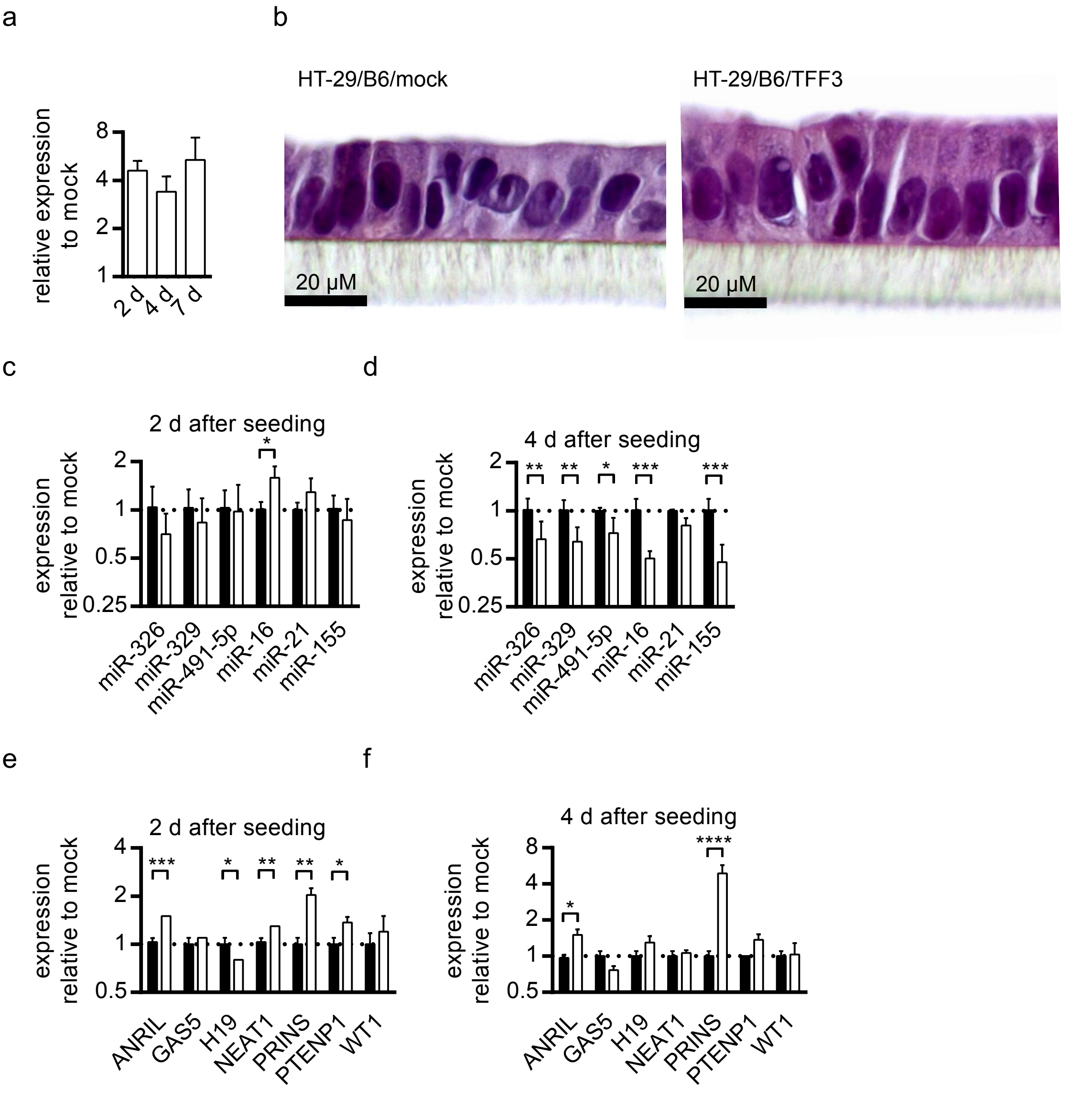

Figure 1: a) TFF3 expression in HT-29/B6/TFF3 compared with mock-transfected controls at days 2, 4 and 7 after seeding. b) H&E staining of differentiated HT-29/B6 subclones at 7 days after seeding. c & d) Expression of miRNAs in HT-29/B6/TFF3 (white columns) compared with HT-29/B6/mock (black columns) over the course of differentiation at 2 and 4 days after seeding. e & f) Expression of lncRNAs in HT-29/B6/TFF3 (white columns) compared with HT-29/B6/mock (black columns) over the course of differentiation at 2 and 4 days after seeding.

Table 1: miRNA targets identified by miRmap analysis (miRmap Score 99).

hsa-miR-16-5p	hsa-miR-21-5p	hsa-miR-155-5p	hsa-miR-326	hsa-miR-329	hsa-miR-491-5p
RNF24	RP11-766F14.2	MARCH1	SBK1	DPY19L3	AEN
CBX6	RAB22A	ZMYM2	SRGAP3	SOX17	SESN2
CACNA2D1	BMPR2	PAQR3	ZC3H12B	DSTYK	MTMR2
KIAA1432		KPNA5	ZZEF1	SRGAP3	PBX2
PRDM5		NHLRC2	TSPAN14	PIK3C3	RHPN1
WDTC1		ZNF652	AMOTL1	ASH1L	PFKFB2
ATXN7L1			LRRC55	HLF	RAP1GAP2
C17orf51			SYT9	CA8	RABL2B
ZBTB34			CBL	DTNA	CCL22
ATG4B			C16orf72	PRDM5	PPP1R18
TMCC1			PRICKLE2	CDCA4	PPP1R3B
MYO5B			C17orf51	TMEM2	CREB3L2
PLAG1			SUDS3	CBL	ZMYM4
MAPK9			STK35	SYT2	DOT1L
CHD2			KIAA0141	PTPLB	FIBCD1
RAB3IP			NCR3LG1	CTTNBP2NL	GFOD1
SREK1			C1orf95	CD2AP	CBX6
TMEM183A			EIF4EBP2	PLAG1	NPTXR
ATG14			SDK2	FAM46C	GNG13
MAN2A2			RAB11FIP4	KIAA2018	TSC1
TK2			TREML2	INO80D	SRGAP3
ABL2			ANXA11	TMOD2	ATP11A
ANKRD34C			PCNX	ABLIM1	ONECUT3
DESI1			TMEM127	LAMTOR3	SNX33
LIN7A			CELSR1	ABL2	HEATR1
UBE2Q1			ADIPOQ	UGT8	ZDHHC14
IPO9			TMEM164	BRI3BP	C16orf52
LUZP1			NFATC2	NFAT5	Z99715.1
SBNO1			hsa-mir-4763	RNF152	SLIT1
ZMAT3			KIF6	FAM105B	MIER3
SFT2D3			HS3ST1	USP36	TTC38
RUNDC3B			LDOC1L	PPP2R2D	POGK
PISD			BRD3	KLHDC5	POLR3A
PRKAB2			ZNF343	JHDM1D	FAM222A
FAT3			NFIC	SLC35B4	40057
MAFK			KIAA1147	BCLAF1	EEPD1
C2orf42			POLR2J2	MED28	KDM4B
TRIP11			ADAM29	ENPP4	LARP1
DLGAP2			SLC7A1	WDR72	KIAA1614
PTCH1			CSNK1G1	FAM126B	IGFBP5
FGF7			RALGPS2	PCNX	AKNA
EIF2C4			GABRA4	FAT3	TSPAN9
USP15			CCDC149	FSTL3	TUSC5
EIF2C1			RRP8	CBX2	hsa-mir-4763
TACC1			SORD	CPEB4	TTC3
TNRC6B			SOGA1	DCAF5	KIR3DX1
DMPK			SEC14L5	CPA4	TNRC6B
BTLA			C22orf29	ZMAT3	GATAD2B
LAMP3			RIMS4	DNAJC5	HRK
NFATC3			PLXNA4	C16orf52	ARID3B
TFAP2A			HIPK2	NLRP11	KCNMB1
C1QL3			PLEKHG4B	TMEM79	ZBTB4
DZIP1			AL033381.1	TFCP2	MLL2
TLK1			SGPL1	EIF3F	CPNE7
AHCYL2			C15orf38	OAS3	SLC28A3
ATF6			NOS1AP	CREB5	MAVS
THAP6			PRKCA	SBNO1	STMN3
KDSR			MGLL	PLCXD1	RNASE13
SOGA1			RFT1	TNRC6B	METTL21A
MCFD2			PRR14L	SMCHD1	CABP7
KCNC2			SHISA7	DLGAP4	MTAP
MNT			PRIMA1	FAM219A	SPIB
SYNRG			ORAI2	MCAM	TFIP11
TBL1XR1			TBC1D13	GRIK3	GNB1L
SIK1			ASXL3	TM9SF3	XCR1
HRK			FBXO41	FAM120C	SORCS2
TMEM221			UBE4A	MYSM1	HHLA2
SLC11A2			DGKG	SPOPL	VPS53
CNOT6L			PAX8	PPARGC1A	CSNK1G1
DNAJB4			SAMD4B	HIPK2	SENP2
BTBD9			POTEG	DNM3	SFTPB
TTPAL			LIMD1	BRWD3	ENY2
FOXK1			DNAJC18	PEAK1	SLC46A1
C10orf54			SSH2	STRN	ELN
APLN			KCNA7	ST6GALNAC3	NFIC
HIPK2			LPP	TMEM132B	SLC7A1
SHOC2			TFCP2L1	C16orf53	ICOSLG
C14orf43			PSAPL1	KIAA2022	BTBD9
BCL11B			MAPK1	EAF1	HIPK2
SLC41A2			ARNT2	FIGN	NOVA2
PAFAH1B1			SH3PXD2A	FOXK1	FOXP4
ACVR2A			WDFY1	STK36	ITGAX
ITGA2			SLC7A11	VANGL1	ARRB1
DGCR14			OXSR1	MAP3K2	RRP7A
RWDD1			PIP5K1C	CDS2	RPS24
THSD4			WSCD1	ZNF571	SLC25A45
MAMSTR			STX6	NOS1AP	ZSCAN12
TUBA4A			TOMM40L	KLF11	HS6ST1
CD40			PDXK	FAM46A	PAX5
RFT1			DNAJB12	GPX6	KALRN
ELL			UBXN10	HIPK1	ADAMTSL1

TMEM189-UBE2V1			H6PD	OCLN	SOX12
TMEM189			LRRC15	PTCHD1	SPOCK2
FGFR1			DHX33	KIAA2026	SEMA6A
SLC1A2			IGF2BP1	C1orf216	MOB3B
TRIM66			BAHD1	C21orf91	FAM212B
FGF2			ELK4	PIK3R1	CDS2
AXIN2			FZD5	MMS22L	C14orf43
QSOX1			CNNM1	LCORL	EGR3
RPRD1B			FAM26E	KPNA4	TNS1
IVNS1ABP			PPM1F	AK4	SLC29A4
TRPC1			MOB3C	SLITRK4	PADI2
HAUS3			CMKLR1	NECAB1	TNNI1
PDK4			PRND	ATP9A	DUSP3
PPT2			SHISA9	NFASC	VWA1
ARHGAP35			EHD3	REEP3	SYT7
STK38L			GRIN2A	METTL9	SHISA6
SSR1			TMEM194B	PAIP2B	PRIMA1
MCU			YY1	ABI2	CHRNB2
ZBTB46			KCNJ5	EPHA4	TNFAIP1
STXBP3			CERS6	MBOAT2	SIRPB2
COL12A1			ARC	FAM204A	ZC3H7B
SLC13A3			MYO9A	ZNF264	HPCAL4
UNC119B			TNFRSF11A	CSRNP3	RP3-324N14.2
NUP50			POTEM	CNOT6	ADAM23
KIAA0317			CBFA2T2	U47924.25	DNAJB5
ZNF609			USH1G	AP000708.1	CLIP2
PAQR3			LONRF2	BACH2	ABCG4
ZMYM2			ZHX3	LANCL3	STX1B
POLR1A			GFRA1	AFF1	CBLN3
INSR			IQSEC3	STX1B	HSPG2
LSM11			PPP1R12B	RTF1	SAMD4B
UBE2V1			C22orf46	GLRA3	EIF5AL1
PHF19			TMEM178B	ZFX	VANGL2
SLC9A8				TFAM	TSKU
UNC80				RNF38	KIAA1161
ARL10				CPSF6	KCNQ4
LIMD1				CCDC71L	DIRAS1
MTHFR				UBE2H	CMIP
DRD1				SLC6A19	FBXO41
CCNJL				EPG5	ARNT2
DCUN1D1				SLC7A11	HOXC12
CCNT1				RAP2B	MAPK1
RBM12				VAPB	SLC38A7
PID1				ZC3H12C	C11orf45
FASN				POU2F1	KCNAB2
CYB5B				SSBP2	ZNF609
PTCD3				MON2	SYNGAP1
VAPB				SMAD2	SH3PXD2A
SRRM4				TCF21	RAMP2
TGIF2				AGPS	RIMS3
PDCD1				SMAD4	ZCCHC24
C5orf64				ZNF304	TBR1
NUCKS1				STRBP	LYPLA2
C18orf1				FAM26E	KCNJ8
ANO3				EDNRA	EPB41L1
CD80				GNAI1	ANKRD52
TBC1D16				ABCC6	PATL1
RASGEF1B				TCF4	TEAD3
POU2F1				HSPA4	HIC2
ASB1				ELK4	GSX1
PTPN4				BLCAP	PEA15
GFAP				CPLX2	FLT4
PRTG				CDK6	ZNF516
AL158821.1				GADL1	NMT1
TMEM43				GLCCI1	ASTN1
RPS6KA3				NHLRC2	CTD-2616J11.4
WNT7A				PALM2-AKAP2	TCEB3
SLC9A6				WIPF2	GIT1
ARFGAP2				RSF1	CDC42BPA
E2F7				MACC1	AR
C14orf37				LNPEP	PDXK
LATS1				TP53INP1	TMEM104
CLCN5				NKTR	SLC6A17
ARIH1				PDE5A	NF2
FOSL2				LARP4	HNRNPUL1
NCS1				RABGAP1	L1CAM
PVRL1				TMEM178B	OPTC
RAB30				IYD	RABL2A
EDA				ATXN1	ENTPD2
ABHD2				IMPAD1	ARHGEF11
FAM123B				PDE3A	N4BP3
ENTPD1				AMMECR1	PPP1R10
ADAMTS12				TNFRSF21	EHD2
HTR2A				PCGF3	TRIOBP
IYD				IGF1R	C10orf105
SNRNP48				PRRG1	ALPK3
PTPN3				MGAT4A	AGPAT1
RAB11FIP2				SLC44A1	AKT2
WEE1				AKAP11	PVRL1
WNK3				NUFIP2	CPLX2
NRP2					ZNF703
IGF1R					KIF21B
TECPR2					IGF2BP1
PPAPDC2					ONECUT2
PPM1L					HMX1
GRM1					SLC6A4

DCLK1					UBTF
HS3ST5					MRPL22
ZFYVE20					C1orf21
CLOCK					SEMA4G
TBRG1					RGS5
PPP1R12B					GPR153
BCL2L2					ALPI
PAPPA					GLP2R
KIAA0247					PLA2G2D
PLSCR4					DRP2
IP6K1					TXNL1
CYBASC3					ZDHHC3
TRANK1					MBD2
					PRDM16
					IQSEC3
					SLIT3
					MEF2D
					DUSP8
					ARF3
					GRAMD1B
					IGSF9B
					USP13
					ELAVL3
					SLAMF8
					NACC1
					SLC25A23

Table 2: DAVID analysis, functional annotation of miR-16, miR-21, miR-155, miR-326, miR-329 & miR-491 targets.

Term	Count	%	PValue	Genes	List Total	Pop Hits	Pop Total	Fold Enrichment	Bonferroni	Benjamini	FDR\
hsa05200:Pathways in cancer	22	3,475513428	0,004232701	PRKCA, FGFR1, AR, FGF7, STK36, CBL, ARNT2, SMAD4, ITGA2, CDK6, SMAD2, FZD5, MAPK1, IGF1R, PAX8, MAPK9, PTCH1, AXIN2, FGF2, WNT7A, PIK3R1, AKT2	177	328	5085	1,926932617	0,416490107	0,164366053	4.8000775655472\
hsa04310:Wnt signaling pathway	16	2,52764613	1,98E-04	PRKCA, TBL1XR1, VANGL1, VANGL2, SMAD4, SMAD2, FZD5, SENP2, PRICKLE2, NFAT5, MAPK9, SOX17, AXIN2, NFATC2, WNT7A, NFATC3	177	151	5085	3,044112695	0,024836372	0,024836372	0.2293947643564942\
hsa04010:MAPK signaling pathway	16	2,52764613	0,042239183	PRKCA, FGFR1, CACNA2D1, FGF7, MAPK1, RPS6KA3, DUSP3, ELK4, ARRB1, MAP3K2, MAPK9, NFATC2, DUSP8, PLA2G2D, FGF2, AKT2	177	267	5085	1,721576843	0,99583469	0,344011919	39.37704687205608\
hsa04144:Endocytosis	14	2,211690363	0,010805424	GIT1, DNM3, ARFGAP2, CBL, PIP5K1C, ZFYVE20, RAB11FIP4, IGF1R, RAB11FIP2, ARRB1, RAB22A, IQSEC3, EHD2, EHD3	177	184	5085	2,185887988	0,748360265	0,205434197	11.837905205473731\
hsa04360:Axon guidance	13	2,05371248	0,001553843	ABLIM1, GNAI1, L1CAM, SLIT1, SLIT3, SEMA6A, EPHA4, MAPK1, SEMA4G, NFAT5, SRGAP3, NFATC2, NFATC3	177	129	5085	2,895151754	0,179212901	0,094026988	1.7872382072132265\
hsa04910:Insulin signaling pathway	12	1,895734597	0,006717384	MAPK1, PPP1R3B, TSC1, PRKAB2, CBL, FASN, MAPK9, PDE3A, INSR, PPARGC1A, PIK3R1, AKT2	177	135	5085	2,553672316	0,57513595	0,192648798	7.518792826147158\
hsa05210:Colorectal cancer	9	1,421800948	0,008126379	IGF1R, MAPK1, SMAD4, MAPK9, SMAD2, AXIN2, FZD5, PIK3R1, AKT2	177	84	5085	3,078087167	0,645223186	0,187186242	9.028789983429174\
hsa04660:T cell receptor signaling pathway	9	1,421800948	0,032697031	MAPK1, CBL, NFAT5, MAPK9, NFATC2, NFATC3, PDCD1, PIK3R1, AKT2	177	108	5085	2,394067797	0,985329661	0,344392348	31.990793922795746\
hsa05218:Melanoma	8	1,263823065	0,010861274	IGF1R, FGFR1, MAPK1, FGF7, CDK6, FGF2, PIK3R1, AKT2	177	71	5085	3,237049415	0,750158206	0,179738825	11.895613220909096\
hsa04370:VEGF signaling pathway	8	1,263823065	0,014448871	PRKCA, MAPK1, NFAT5, NFATC2, PLA2G2D, NFATC3, PIK3R1, AKT2	177	75	5085	3,06440678	0,84250988	0,206299481	15.531098437996116\
hsa04914:Progesterone-mediated oocyte maturation	8	1,263823065	0,028546524	IGF1R, MAPK1, RPS6KA3, GNAI1, MAPK9, PDE3A, PIK3R1, AKT2	177	86	5085	2,672447773	0,974730606	0,335476206	28.528603158848597\
hsa04540:Gap junction	8	1,263823065	0,033605109	PRKCA, MAPK1, DRD1, GNAI1, MAP3K2, TUBA4A, GRM1, HTR2A	177	89	5085	2,582365264	0,986979207	0,326087668	32.72753830384178\
hsa05215:Prostate cancer	8	1,263823065	0,033605109	IGF1R, FGFR1, MAPK1, AR, CREB3L2, CREB5, PIK3R1, AKT2	177	89	5085	2,582365264	0,986979207	0,326087668	32.72753830384178\
hsa04270:Vascular smooth muscle contraction	8	1,263823065	0,092217663	PRKCA, EDNRA, MAPK1, RAMP2, PPP1R12B, PLA2G2D, KCNMB1, ARHGEF11	177	112	5085	2,052058111	0,99999539	0,459016554	67.4379576306945\
hsa05212:Pancreatic cancer	7	1,105845182	0,037438969	MAPK1, SMAD4, MAPK9, CDK6, SMAD2, PIK3R1, AKT2	177	72	5085	2,793079096	0,992140571	0,332246895	35.758342494522765\
hsa04520:Adherens junction	7	1,105845182	0,049410736	IGF1R, FGFR1, MAPK1, PVRL1, SMAD4, SMAD2, INSR	177	77	5085	2,611710324	0,998396389	0,368513298	44.43741714216388\
hsa04012:ErbB signaling pathway	7	1,105845182	0,079737075	PRKCA, MAPK1, CBL, MAPK9, ABL2, PIK3R1, AKT2	177	87	5085	2,311513735	0,99997389	0,462472291	61.85088371616558\
hsa05214:Glioma	6	0,947867299	0,065784631	PRKCA, IGF1R, MAPK1, CDK6, PIK3R1, AKT2	177	63	5085	2,736077482	0,99982349	0,43793743	54.57737681746657\
hsa00564:Glycerophospholipid metabolism	6	0,947867299	0,085236782	CDS2, DGKG, LYPLA2, PISD, PLA2G2D, AGPAT1	177	68	5085	2,534895314	0,999987804	0,466651182	64.41273230531918\
hsa04730:Long-term depression	6	0,947867299	0,089454964	PRKCA, IGF1R, MAPK1, GNAI1, PLA2G2D, GRM1	177	69	5085	2,498157701	0,999993219	0,465482168	66.27000267451388\
hsa04930:Type II diabetes mellitus	5	0,789889415	0,078195247	MAPK1, MAPK9, INSR, ADIPOQ, PIK3R1	177	47	5085	3,056256762	0,999967704	0,476011674	61.10303226748772\

Table 3: DAVID analysis, functional annotation of miR-326, miR-329 & miR-491 targets.

Term	Count	%	PValue	Genes	List Total	Pop Hits	Pop Total	Fold Enrichment	Bonferroni	Benjamini	FDR\
hsa05200:Pathways in cancer	14	3,03030303	0,058079835	PRKCA, AR, STK36, CBL, ARNT2, SMAD4, SMAD2, CDK6, FZD5, IGF1R, MAPK1, PAX8, PIK3R1, AKT2	126	328	5085	1,722560976	0,998529259	0,352605379	49.07871667024287\
hsa04360:Axon guidance	12	2,597402597	2,90E-04	ABLIM1, EPHA4, MAPK1, SEMA6A, SEMA4G, GNAI1, SRGAP3, NFAT5, L1CAM, NFATC2, SLIT1, SLIT3	126	129	5085	3,754152824	0,03112607	0,03112607	0.32667407699999984 \
hsa04310:Wnt signaling pathway	11	2,380952381	0,003761237	PRKCA, SENP2, VANGL1, VANGL2, PRICKLE2, NFAT5, SMAD4, SMAD2, SOX17, NFATC2, FZD5	126	151	5085	2,939924314	0,336845832	0,185657217	4.1613168217404795\
hsa04144:Endocytosis	10	2,164502165	0,036395256	RAB11FIP4, GIT1, DNM3, IGF1R, ARRB1, CBL, PIP5K1C, IQSEC3, EHD2, EHD3	126	184	5085	2,193322981	0,98242144	0,332427668	34.17474750466888\
hsa04270:Vascular smooth muscle contraction	8	1,731601732	0,019700972	PRKCA, EDNRA, MAPK1, RAMP2, PPP1R12B, PLA2G2D, KCNMB1, ARHGEF11	126	112	5085	2,882653061	0,885689962	0,30335093	20.102817779701667\
hsa04530:Tight junction	8	1,731601732	0,04603243	PRKCA, EPB41L1, OCLN, GNAI1, ASH1L, AMOTL1, PPP2R2D, AKT2	126	134	5085	2,409381663	0,994122893	0,326407563	41.23007096868153\
hsa04910:Insulin signaling pathway	8	1,731601732	0,047595708	MAPK1, PPP1R3B, TSC1, CBL, PDE3A, PPARGC1A, PIK3R1, AKT2	126	135	5085	2,391534392	0,995084966	0,315916626	42.307235574911154\
hsa04370:VEGF signaling pathway	7	1,515151515	0,009873027	PRKCA, MAPK1, NFAT5, NFATC2, PLA2G2D, PIK3R1, AKT2	126	75	5085	3,766666667	0,660916073	0,302674199	10.5878301348873\
hsa05210:Colorectal cancer	7	1,515151515	0,016679112	IGF1R, MAPK1, SMAD4, SMAD2, FZD5, PIK3R1, AKT2	126	84	5085	3,363095238	0,84012379	0,367666835	17.2804226791916\
hsa05215:Prostate cancer	7	1,515151515	0,021604876	IGF1R, MAPK1, AR, CREB3L2, CREB5, PIK3R1, AKT2	126	89	5085	3,174157303	0,907518156	0,288305216	21.83568895241311\
hsa05214:Glioma	6	1,298701299	0,018685323	PRKCA, IGF1R, MAPK1, CDK6, PIK3R1, AKT2	126	63	5085	3,843537415	0,872031196	0,337141511	19.16415552442352\
hsa05212:Pancreatic cancer	6	1,298701299	0,031274388	MAPK1, SMAD4, CDK6, SMAD2, PIK3R1, AKT2	126	72	5085	3,363095238	0,968675422	0,351386993	30.11956032439094\
hsa04070:Phosphatidylinositol signaling system	6	1,298701299	0,03465065	PRKCA, CDS2, DGKG, PIK3C3, PIP5K1C, PIK3R1	126	74	5085	3,272200772	0,978590259	0,347602362	32.81795000096294\
hsa05220:Chronic myeloid leukemia	6	1,298701299	0,03642048	MAPK1, CBL, SMAD4, CDK6, PIK3R1, AKT2	126	75	5085	3,228571429	0,982471525	0,3076266	34.19417989075774\
hsa04914:Progesterone-mediated oocyte maturation	6	1,298701299	0,05956259	IGF1R, MAPK1, GNAI1, PDE3A, PIK3R1, AKT2	126	86	5085	2,815614618	0,998761321	0,341872677	49.97557269902019\
hsa04012:ErbB signaling pathway	6	1,298701299	0,062003625	PRKCA, MAPK1, CBL, ABL2, PIK3R1, AKT2	126	87	5085	2,783251232	0,999066904	0,336623046	51.42074376050009\
hsa04666:Fc gamma R-mediated phagocytosis	6	1,298701299	0,083541552	PRKCA, DNM3, MAPK1, PIP5K1C, PIK3R1, AKT2	126	95	5085	2,54887218	0,999925819	0,410380515	62.61806123351181\
hsa05223:Non-small cell lung cancer	5	1,082251082	0,042819904	PRKCA, MAPK1, CDK6, PIK3R1, AKT2	126	54	5085	3,736772487	0,991521699	0,328015789	38.95875819096904\
hsa00564:Glycerophospholipid metabolism	5	1,082251082	0,085227741	CDS2, DGKG, LYPLA2, PLA2G2D, AGPAT1	126	68	5085	2,967436975	0,99993931	0,400127839	63.386539134450274\
hsa04730:Long-term depression	5	1,082251082	0,088834843	PRKCA, IGF1R, MAPK1, GNAI1, PLA2G2D	126	69	5085	2,924430642	0,999960547	0,397711993	64.98235163378081\
hsa05218:Melanoma	5	1,082251082	0,096267041	IGF1R, MAPK1, CDK6, PIK3R1, AKT2	126	71	5085	2,842052314	0,999983843	0,408673564	68.07234673725269\

Table 4: Pubmed IDs of relevant literature taken as a basis for the selection.

**miRNAs**
miR-326: 25760058, 25138213, 25087086, 23869222, 23522246, 23341351, 20667897, 19955368<br>miR-329: 26337669, 26320179, 24944161, 24423585<br>miR-491: 26034994, 25299770, 24148764, 23519249, 21373755, 20039318
**lncRNAs**
ANRIL: 25636450<br>GAS5: 24884417<br>H19: 22776265<br>NEAT1: 25417700<br>PRINS: 20377629<br>PTENP1: 20577206<br>WT1: 26071132

RNAhybrid parameters: default with mfe ≤ -20 kcal/mol and helix constraint from 2-8.

hsa-miR-155
```
No interaction.
```

hsa-miR-326
```
binding site #1: position 1754
mfe: -21.1 kcal/mol
PRINS (HG975433.1) 5'  A    U  CCUGAUUCU    UUUCCCUGCCCCC       U 3'
                        GGAG GG         GGGG             CCUGGGG
                        CCUC CU         UCCC             GGGUCUC
hsa-miR-326        3' GA                                        C 5'
```

hsa-miR-329
```
binding site #1: position  1082
mfe: -27.4 kcal/mol
PRINS (HG975433.1) 5'     A    U     AU        A 3'
                           GGAA CCCAG  AGGACCUC
                           UCUU GGGUC  UUUUGGAG
hsa-miR-329        3' CUUUG    U                 5'
hsa-miR-329
binding site #2: position  584
mfe: -25.0 kcal/mol
PRINS (HG975433.1) 5' U         GAAUGGCAUGA      GAGGU       G 3'
                       GAGGCAGGA           ACCCGG     GGAGCUU
                       CUUUGUCUU           UGGGUC     UUUUGGA
hsa-miR-329        3'                                        G 5'
```

hsa-miR-491-5p
```
binding site #1: position  1552
mfe: -27.5 kcal/mol
PRINS (HG975433.1)    5'  G        C   CGGG      C 3'
                             UUGUGGAG GGG    UCCCGC
                             AGUACCUU CCC    GGGGUG
hsa-miR-491-5p        3' GG            AA        A 5'
binding site #2: position  1761
mfe: -26.8 kcal/mol
PRINS (HG975433.1)    5' G   G  UCU          U   3'
                          CCU AU   GGGGUUUCCC
                          GGA UA   UCCCAAGGGG
hsa-miR-491-5p               3' G      CCU          UGA 5'
binding site #3: position  183
mfe: -23.6 kcal/mol
PRINS (HG975433.1)    5' U    UUUA    C  GG       G 3'
                          UCUU    GAGG GG  UCUCACU
                          GGAG    CUUC CC  GGGGUGA
hsa-miR-491-5p        3'      UAC        AA         5'
```

Figure 2: RNAHybrid analysis of PRINS interaction with miR-155, miR-326, miR-329 & miR-491.

```
Binding site #1:
GGCCGCggacgagctggttgtggagcgggcgggtcccgccacccgtgggcT
    CGcctgctcgaccaacacctcgcccgcccagggcggtgggcacccgAGATC

Binding site #2:
GGCCGCtacaggagtggcctgattctggggtttccctgccccccctgT
    CGatgtcctcaccggactaagaccccaaagggacggggggacAGATC

Binding site #3:
GGCCGCtttttaaatttctttttagaggcggggtctcactgtgttttgcccT
    CGaaaaaatttaaagaaaaatctccgccccagagtgacacaaaacgggAGATC

Binding site #3m:
GGCCGCtttttaaatttctttttagCCCTTTCACTATCTGCtgttttgcccT
    CGaaaaaatttaaagaaaaatcGGGAAAGTGATAGACGacaaaacgggAGATC
```

Figure 3: Luciferase reporter constructs for interaction studies; hsa-miR-491-5p binding-sites cloned in pTK-Gluc (NotI/XbaI). Yellow marks show predicted binding-sites and red letters depict the mutated site.

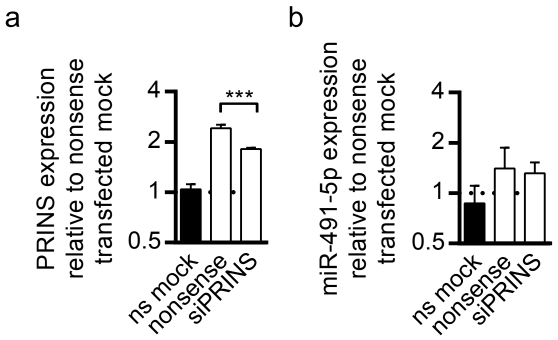

Figure 4: a) Knock down efficiency of siRNA vs. PRINS in HT-29/B6/TFF3. b) Expression of miR-491-5p after specific knockdown of PRINS. Black columns show nonsense transfected mock controls and white columns HT-29/B6/TFF3 cells.

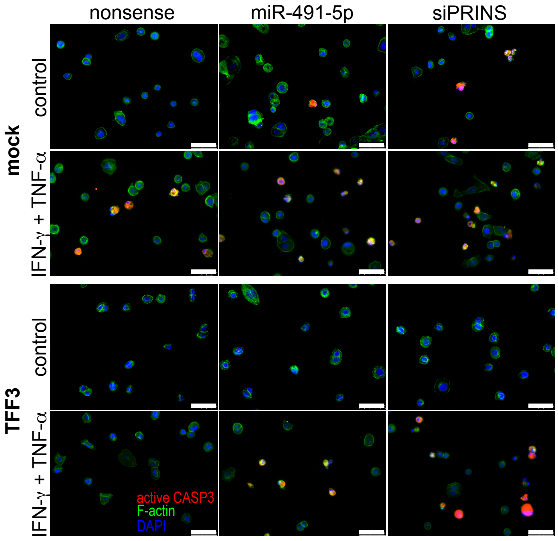

Figure 5: Transfected cells were seeded at reduced density and apoptotic cells (stained for active caspase-3, stained red) were counted automatically using ImageJ (96 images, 8 replicates per treatment). Nuclei were stained with DAPI (blue) and F-actin was stained with Phalloidin-conjugates (green).

Table 5: Sequence and position of Stellaris FISH probes against PRINS

PRINS Probe	Sequence (5'-3')	Position
1	tgcacatggctttttataca	345
2	cctctctctgatttattgga	710
3	tgtcctcattagtcttctag	732
4	cagggcaaccatttctgttt	839
5	cctcccttgaaagaacacag	868
6	gtgtgtgagatgaaggtacc	914
7	tcaaagcacaaatgcacgcc	941
8	ccaagaagtgactgatccta	1019
9	gccactggaaatgagactac	1046
10	attccttttgccaagggaag	1068
11	gtttagaaagatgaggtcct	1095
12	gcaaacatgggatggttagg	1131
13	gttgataattccaggcaact	1165
14	gagtcaaaagatgccctgac	1207
15	cttttccgtagttctcactg	1353
16	gggtttaggtaaatccggat	1406
17	tactatgcaaatgccacctg	1430
18	cacagctagaaaataccggt	1457
19	cgctcaggatttgaagtgtg	1480
20	ttaatgttctgggagcagtc	1595
21	gatgtggcatggaacatcag	1623
22	cagacatgggtcttcatgtc	1645
23	ctttggcccttctacagaag	1667
24	cagggaaaccccagaatcag	1763
25	tcacacagcagtttagggac	1794
26	ggaacaactgactcttgggt	1840
27	gaaagaaaccagattggccc	1899
28	tcattctctgcttgagaagc	1931
29	caaggtttaactgctgtggg	1954
30	accatacttagaaacatcct	2062
31	ggcaaggtaagatgaactga	2086
32	cacactcattctttcagagc	2108
33	ggcatttctaaatctgatcc	2130
34	cactcggctttctaaccttt	2152
35	ctgagcttgagttaatcggc	2174

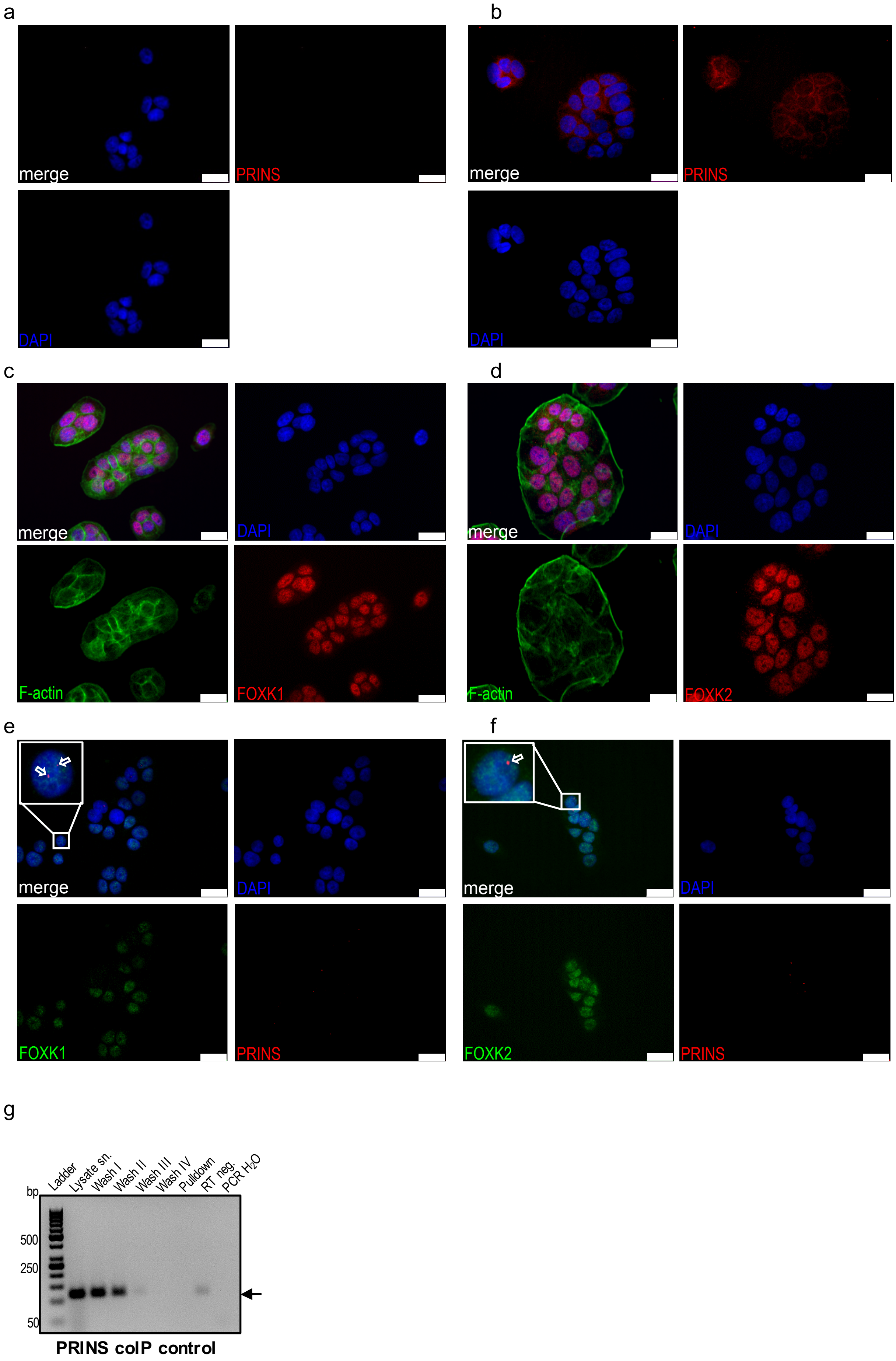
a
b
merge
PRINS
DAPI
c
d
merge
DAPI
F-actin
FOXK1
FOXK2
e
f
FOXK1
FOXK2
g
Ladder
Lysate sn.
Wash I
Wash II
Wash III
Wash IV
Pulldown
RT neg.
PCR H2O
bp
500
250
50
PRINS coIP control

Figure 6: Controls for PRINS-specific Stellaris FISH and co-localisation studies with FOXK1 and 2; a) Stellaris FISH without probes; b) Stellaris FISH after RNase treatment with probes, in RNase treated controls focal signals disappeared and a scattered PRINS signal remained due to potential binding of probes to PRINS artefacts; c) FOXK1 IF detection; d) FOXK2 IF detection; e) Co-localisation of FOXK1 and PRINS; f) Co-localisation of FOXK2 and PRINS. g) CoIP control experiments using mormal rabbit IgG instead of PMAIP1-specific antibody proved absence of non-specific PRINS binding in pulldown samples.
